# Empiric treatment of pulmonary TB in the Xpert era: Correspondence of sputum culture, Xpert MTB/RIF, and clinical diagnoses

**DOI:** 10.1371/journal.pone.0220251

**Published:** 2019-07-24

**Authors:** Emily A. Kendall, Caleb Kamoga, Peter J. Kitonsa, Annet Nalutaaya, Phillip P. Salvatore, Katherine Robsky, Olga Nakasolya, James Mukiibi, David Isooba, Adithya Cattamanchi, Midori Kato-Maeda, Achilles Katamba, David W. Dowdy

**Affiliations:** 1 Division of Infectious Diseases and Center for Tuberculosis Research, Department of Medicine, Johns Hopkins University, Baltimore, MD, United States America; 2 Uganda Tuberculosis Implementation Research Consortium, Makerere University, Kampala, Uganda; 3 Department of Epidemiology, Johns Hopkins Bloomberg School of Public Health, Baltimore, MD, United States America; 4 Division of Pulmonary, Critical Care, Allergy and Sleep Medicine, Department of Medicine, University of California San Francisco, San Francisco, CA, United States America; 5 Department of Medicine, College of Health Sciences, Makerere University, Kampala, Uganda; Wadsworth Center, UNITED STATES

## Abstract

**Background:**

Clinical tuberculosis diagnosis and empiric treatment have traditionally been common among patients with negative bacteriologic test results. Increasing availability of rapid molecular diagnostic tests, including Xpert MTB/RIF and the new Xpert Ultra cartridge, may alter the role of empiric treatment.

**Methods:**

We prospectively enrolled outpatients age > = 15 who were evaluated for pulmonary tuberculosis at three health facilities in Kampala, Uganda. Using sputum mycobacterial culture, interviews, and clinical record abstraction, we estimated the accuracy of clinical diagnosis relative to Xpert and sputum culture and assessed the contribution of clinical diagnosis to case detection.

**Results:**

Over a period of 9 months, 99 patients were diagnosed with pulmonary tuberculosis and subsequently completed sputum culture; they were matched to 196 patients receiving negative tuberculosis evaluations in the same facilities. Xpert was included in the evaluation of 291 (99%) patients. Compared to culture, Xpert had a sensitivity of 92% (95% confidence interval 83–97%) and specificity of 95% (92–98%). Twenty patients with negative Xpert were clinically diagnosed with tuberculosis and subsequently had their culture status determined; two (10%) were culture-positive. Considering all treated patients regardless of Xpert and culture data completeness, and considering treatment initiations before a positive Xpert (N = 4) to be empiric, 26/101 (26%) tuberculosis treatment courses were started empirically. Compared to sputum smear- or Xpert-positive patients with positive cultures, empirically-treated, Xpert-negative patients with negative cultures had higher prevalence of HIV (67% versus 37%), shorter duration of cough (median 4 versus 8 weeks), and lower inflammatory markers (median CRP 7 versus 101 mg/L).

**Conclusion:**

Judged against sputum culture in a routine care setting of high HIV prevalence, the accuracy of Xpert was high. Clinical judgment identified a small number of additional culture-positive cases, but with poor specificity. Although clinicians should continue to prescribe tuberculosis treatment for Xpert-negative patients whose clinical presentations strongly suggest pulmonary tuberculosis, they should also carefully consider alternative diagnoses.

## Introduction

Empiric treatment for tuberculosis (TB), in the absence of bacteriologic confirmation, has long been a common practice made necessary by the limited sensitivity of available diagnostic assays [1,2]. Conventional diagnosis by sputum smear microscopy misses 40–50% of symptomatic, culture-positive pulmonary TB [3]. Lack of confidence in assay sensitivity often leads clinicians to err on the side of TB treatment for high-risk patients. Xpert MTB/RIF (Xpert), a rapid molecular diagnostic test for TB introduced in 2011, has improved diagnostic sensitivity, and the next-generation Xpert MTB/RIF Ultra cartridge (plus other fast-followers) should improve this sensitivity still further. Still, compared to sputum culture, the false-negative rate of Xpert–and even of Xpert Ultra–remain higher than the 5% which was identified in a WHO-led Delphi consensus process as the target for a sputum-based TB diagnostic assay [4].

Xpert has led to more bacteriologic confirmation among patients treated for TB [5], but it has been more difficult to observe the effect of Xpert on the accuracy of clinical diagnosis (i.e., diagnosis made without bacteriological confirmation) [6]. There is some evidence that empiric treatment declines over time after Xpert introduction, as clinicians grow more comfortable with using it to rule out TB [7], but the high prevalence of bacteriologically unconfirmed TB notifications (44% of pulmonary TB notifications worldwide in 2017 [8]) reveals the continued prominent role of clinical diagnosis in many high burden settings. The frequency, accuracy, and contribution to case detection of clinical TB diagnosis in clinical settings which have fully implemented point-of-care Xpert remains uncertain. Better understanding of the role of clinical diagnosis in such settings can be important for Xpert implementation and clinician training.

## Methods

We evaluated the role of clinical diagnosis (i.e. diagnosis without bacteriologic confirmation) among adults being evaluated for pulmonary TB in a real-world high HIV- and TB-burden setting where on-site use of Xpert is well established. We observed the results of Xpert and other diagnostic testing and incorporation of these results into clinicians’ treatment decisions. We subsequently performed sputum culture (as a gold standard), and measured the sensitivity and specificity of Xpert and of the overall clinician treatment decision for culture-positive TB. We report on the additional contribution of clinical diagnoses to TB case detection and on the characteristics of empirically-treated patients whose sputum cultures were ultimately negative for *Mycobacterium tuberculosis*.

### Study setting and population

Participants were enrolled from three health facilities (a large public health center with a TB clinic, and two private nonprofit clinics with a focus on HIV care) that had been identified as the primary clinics providing TB care to residents of neighboring parishes in Kampala, Uganda. The two largest facilities had on-site GeneXpert devices and were preparing to transition to the use of Xpert Ultra cartridges, and the third referred its specimens to nearby clinical or reference laboratories for Xpert.

Between May 2018 and February 2019, we recruited patients aged ≥15 years who were diagnosed with pulmonary TB at these health facilities; eligibility for TB culture was restricted to residents of three parishes nearest the health facilities, except during a six-month period at one health facility when no residence-based inclusion criteria were imposed (in order to expand sample size). For the current analysis, we excluded patients for whom TB treatment was initiated based on a history of partial TB treatment and previous loss to follow-up. For each enrolled case, we randomly selected two controls aged ≥15 years, matched by health facility and residence (within versus outside the three parishes nearest the health facilities), from among those patients who were evaluated for pulmonary TB and determined not to have TB on a selected day following the case’s enrollment. Diagnosis and treatment decisions were made by routine clinical staff, and eligible individuals were subsequently identified by research study staff via review of the health facilities’ presumptive TB registers after decisions to treat or not treat for TB had been made. Health facility clinicians retrospectively confirmed that all enrolled cases had been diagnosed with pulmonary TB and recommended for treatment, and that all controls had been evaluated for suspected pulmonary TB but neither diagnosed with, nor treated for, TB at the end of that evaluation.

### Clinical and laboratory data collection

Patients were invited to enroll in the study on the day that they were informed of the clinical decision for or against a TB diagnosis. After providing written informed consent (or for adolescents, assent and parent/guardian assent), enrolled cases and controls participated in a standardized interview that included TB symptoms, risk factors, and care seeking. They provided expectorated sputum for mycobacterial culture (described below) and venous blood for CRP measurement (i-CHROMA, Boditech, Korea) at the time of study enrollment. Neither test was part of standard care; the tests were performed after clinician decisions to treat or not treat for TB, and clinicians were not informed of culture or CRP results except in instances when an enrolled control (a patient not treated for TB) had a positive sputum culture result. Medical records were abstracted to collect information about the TB evaluation including any Xpert or other diagnostic testing, any HIV and ART history, and results of HIV testing which was routinely performed as part of TB diagnostic evaluation.

For culture, a single expectorated sputum was collected from each participant and subjected to *N*-acetyl-l-cysteine–sodium hydroxide (NALC-NaOH) digestion and decontamination [9]. The sediment was split for liquid culture using the BACTEC MGIT 960 system (BD Diagnostics, USA) over 42 days, and solid culture on Lӧwenstein-Jensen (LJ) media held at 37°C for 56 days. Growth was confirmed as *M*. *tuberculosis* by Ziehl-Neelsen stain and by MPT64 antigen immunochromatography (SD Bioline, Standard Diagnostics, Korea). Cultures with non-acid fast bacilli (AFB) growth on blood agar were re-decontaminated using 4% NaOH and re-incubated. Sputum cultures were classified as TB positive if liquid or solid or both culture media grew *M*. *tuberculosis*, undetermined if a patient was unable to produce sputum or both cultures were contaminated, and TB negative otherwise (including specimens with negative solid culture and contaminated liquid culture).

Study data were collected and managed using Research Electronic Data Capture (REDCap) tools hosted at Johns Hopkins University [10].

### Statistical methods

The accuracy of Xpert relative to culture was analyzed only in patients with definitive culture results (excluding those whose culture status could not be determined due to contamination or inability to produce sputum). In sensitivity analyses, we presumed that those patients with missing or contaminated cultures either had (a) culture results that corresponded to their Xpert results or (b) culture results that corresponded to their clnicians’ treatment decisions (positive cultures in those treated, negative cultures in those not treated). Group characteristics were compared using Wilcoxon test for continuous variables or Fisher’s exact test for categorical variables. Sensitivities and specificities including exact 95% binomial confidence intervals were calculated for participants who had definitive Xpert and culture results. Relative risks are reported with 95% Wald confidence intervals. Data analysis was performed using R version 3.5.2 [11] and the R packages *binom*, *epitools*, and *dplyr*.

### Human subjects considerations

The study was approved by the Ethics Review Committee of the Makerere University School of Public Health (Protocol 544). Study participants age 18 years of older gave written informed consent, and participants age 15–17 years provided written assent with written consent of a parent or guardian. Controls with positive sputum cultures were referred to the local TB control program clinics for treatment.

## Results

### Basis for TB diagnosis

We enrolled and attempted sputum culture for 301 participants eligible for this analysis: 101 cases diagnosed with TB, and 200 controls evaluated 0–120 days (median 13 days) after the corresponding case.

Among the 101 cases, sputum of 98 was tested with Xpert, two underwent sputum AFB smear examination, and one had chest x-ray but no sputum testing performed as part of the clinical TB evaluation.

**Fig 1:**
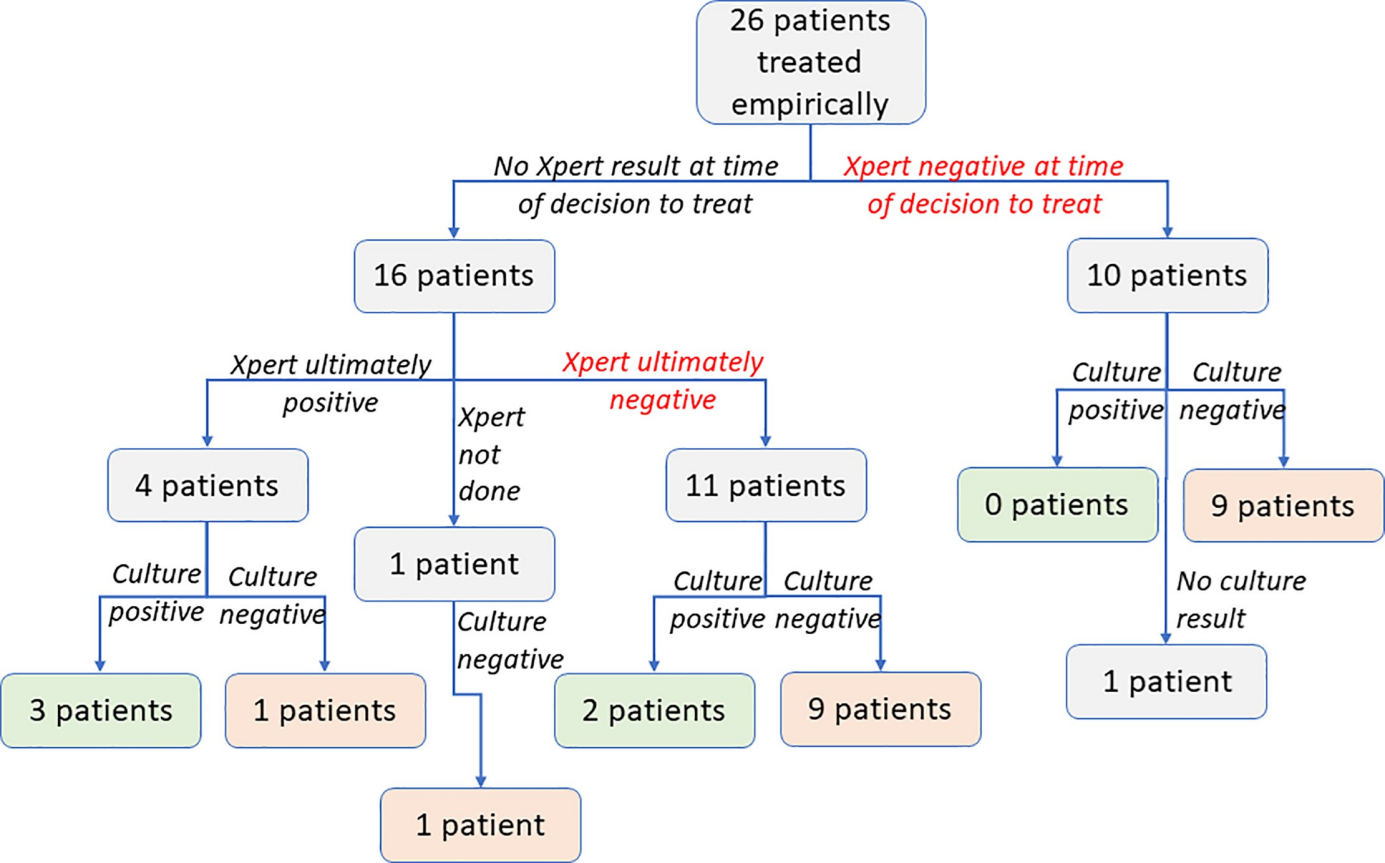
Xpert results at time of diagnosis, and eventual Xpert and sputum culture results, among empirically treated patients. No other bacteriologic testing such as sputum smear examination was used in diagnosing these individuals.

Overall, 77 (76%) cases had a positive Xpert or smear examination, including 4 who were started on treatment empirically before their positive Xpert result returned. The remaining 24 (24%) cases were treated empirically with no Xpert or AFB smear support for the diagnosis of TB. These included those whose tests were negative before the start of treatment, those whose negative test results became available after treatment began, and one for whom sputum testing was never performed (Fig 1).

Of the 200 controls, 199 had sputum Xpert tests performed, all of which were negative. The additional control had a negative AFB sputum smear examination.

Cases were more likely than controls to be male (61% versus 40%, p < 0.001), to have a history of previous TB (23% versus 10%, p = 0.005), to have untreated HIV (15% versus 3%, p < 0.001), and to use alcohol regularly (22% versus 7%, p < 0.001) (Table 1). There were higher proportions of smoking and overall HIV among cases as well (p = 0.1 for each). Distributions of age, household income, and education levels were similar for cases and controls. All participants reported cough, but cases reported a longer median duration of cough (5 versus 2 weeks) and were also more likely to report additional TB symptoms of fever, sweats, or weight loss (p<0.002 for each).

**Table 1:** Characteristics of TB cases and of TB-negative controls, as classified by clinicians after evaluation for presumptive pulmonary TB.

	Cases (N = 101)	Controls (N = 200)
**Age in years (Median, IQR)**	31 (26–38)	32.5 (25–43)
**Male (N, %)**	62 (61%)	79 (40%)
**HIV+ (N, %)**	43 (43%)	66 (33%)
On ART if HIV+ (N, %)*	32 (74%)	62 (95%)
**Prior TB (N, %)**	23 (23%)	20 (10%)
**Current smoker (N, %)**	13 (13%)	14 (7%)
**Alcohol >4 drinks/week (N, %)**	22 (22%)	14 (7%)
**Education beyond primary school (N, %)**	33 (33%)	69 (34%)
**Prior incarceration (N, %)**	44 (44%)	56 (28%)
**Household income (in 1000s UGX; Median, IQR)**	380 (170–600)	330 (200–582.5)
**Reports current cough (N, %)**	101 (100%)	200 (100%)
**Weeks of cough (Median, IQR)**	5 (3–12)	2 (1–4.25)
**Reports current fever (N, %)**	46 (46%)	47 (24%)
**Reports current night sweats (N, %)**	44 (44%)	32 (16%)
**Reports weight loss (N, %)**	74 (73%)	70 (35%)

* ART status is out of 43 HIV+ cases and 65 HIV+ controls with known ART status

### Sputum culture

Of the 99 cases with culture results, 19 started anti-TB treatment before enrolling into the study and providing sputum for culture: 9 received only a single dose before collection of sputum for culture (of which 6 [67%] cultures were positive), 8 received 2–7 days of treatment before culture (2 [25%] cultures positive), and 2 received 8–14 days of treatment before culture (2 [100%] cultures positive).

Culture status could be determined for 293 (97%) study participants: 99 cases (including 96 cases with Xpert results) and 1894 controls (including 184 controls with Xpert results whose matched cases also had definitive culture and Xpert results, thus allowing them to be included in analyses of Xpert sensitivity and clinical judgment relative to culture.)

Unavailable culture results included one case who could not produce additional sputum for culture (but who had a positive sputum Xpert result)and one case and six controls (all Xpert negative) for whom both solid and liquid cultures were contaminated. Liquid culture contamination was common (13%), but solid culture contamination was rare (3%), and there was otherwise good agreement between liquid and solid culture (S1 Table).

Of the 289 patients with a definitive sputum culture result (additionally excluding controls whose matched case did not have a definitive sputum culture result), sputum culture was positive in 71 (72%) of 99 patients enrolled as cases and 4 (2%) of 181 enrolled as controls.

### Accuracy of Xpert and clinical diagnosis, relative to culture

**Fig 2:**
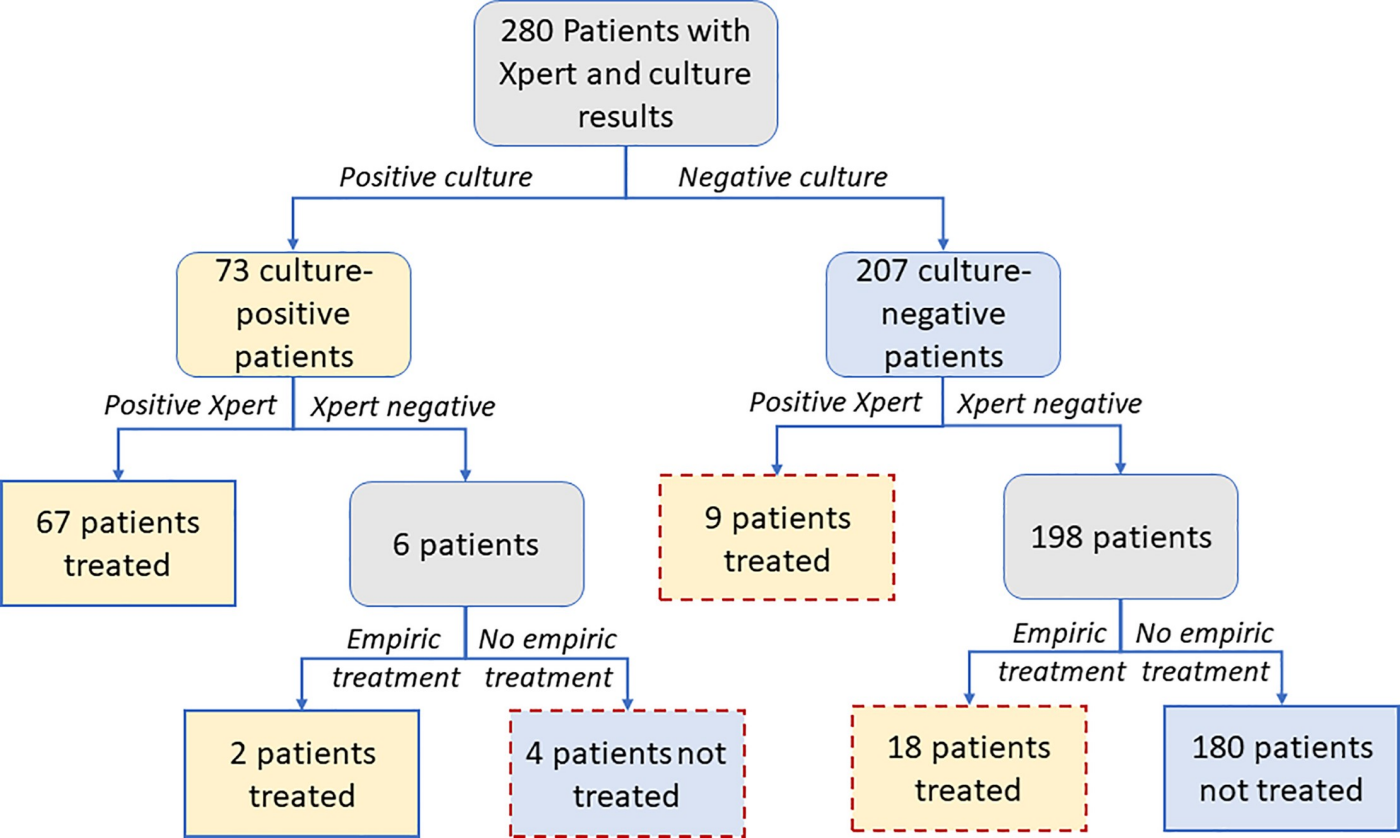
Xpert results and treatment decisions, by sputum culture result. Red dashed outlines highlight the patients for whom culture results were discordant with their previous diagnosis, including 4 with positive culture who had not been treated (false-negative Xpert) and 27 with negative cultures who had been treated either based on Xpert or empirically. For some patients with positive Xpert, the decision to treat was made before the Xpert result became available; these patients are classified as “Positive Xpert” here, but they are also included in Fig 1 which describes all empirically treated cases. Controls were included in this figure only if their matched case also had Xpert and culture results.

Clinicians’ diagnostic decisions (prior to availability of a culture result) and patients’ subsequent culture results are illustrated for all empirically-treated patients with a definitive culture result in Fig 1, and for all patients with definitive Xpert and culture results (and, for controls, whose matched cases also had definitive Xpert and culture results) in Fig 2. Relative to sputum culture, Xpert had a sensitivity of 92% (95% CI 83–97%) and a specificity of 96% (95% CI 92–98%) (Table 2, with results stratified by Ultra versus the earlier (G4) Xpert cartridge in S2 Table and S3 Table). Sensitivity of Xpert relative to sputum culture was lower in people with HIV, and specificity was lower in those with a history of previous TB treatment (Table 2). Clinical diagnosis increased sensitivity of the overall TB evaluation to 95% (95% CI 87–98%), but with substantial reduction in specificity (Table 2).

**Table 2:** Sensitivity and specificity of Xpert and clinical judgment, relative to sputum culture.

	*Sensitivity*			*Specificity*		
	N	Estimate	95% CI	N	Estimate	95% CI
**Xpert, All patients**	67/73	92%	83–97%	198/207	96%	92–98%
**Xpert, HIV+**	25/31	81%	63–93%	66/69	96%	88–99%
**Xpert, Previously treated**	14/14	100%	77–100%	19/22	86%	65–97%
**Xpert + clinical diagnosis, All patients**	69/73	95%	87–98%	180–207	87%	82–91%
**Xpert + clinical diagnosis, HIV+**	27/31	87%	70–96%	54/69	78%	67–87%
**Xpert + clinical diagnosis, Previously treated**	14/14	100%	77–100%	16/22	73%	50–89%

26 (26%) treated patients were treated empirically (Fig 1): 10 were treated after a negative Xpert result had been obtained (of whom nine had negative and one had contaminated cultures), and the other 16 were treated before their Xpert results became available. Of the 16 patients empirically treated before an Xpert result, four had positive Xpert results reported after treatment initiation (of whom three were culture-positive, and the fourth, who had a history of prior TB treatment, was culture-negative); one was treated based on chest x-ray with no Xpert performed (and was culture negative), and the remaining eleven had negative Xpert results (of whom two were culture-positive and nine were culture-negative). Overall, of 25 empirically-treated patients with definitive culture results (i.e. excluding one patient with contaminated cultures), five (20%) had positive cultures for TB, including three (12%) who had positive Xpert results obtained after the start of treatment. Thus, of 20 empirically treated patients with Xpert-negative sputum and a definitive culture result, two (10%) had a positive sputum culture.

In sensitivity analysis, there was minimal change in the estimated sensitivity and specificity of Xpert if all patients with contaminated or missing cultures (N = 7) were assumed to have culture result that corresponded to their Xpert results (S4 Table) or to their clinicians’ treatment decisions (S5 Table).

### Characterizing culture-discrepant diagnoses

**Fig 3:**
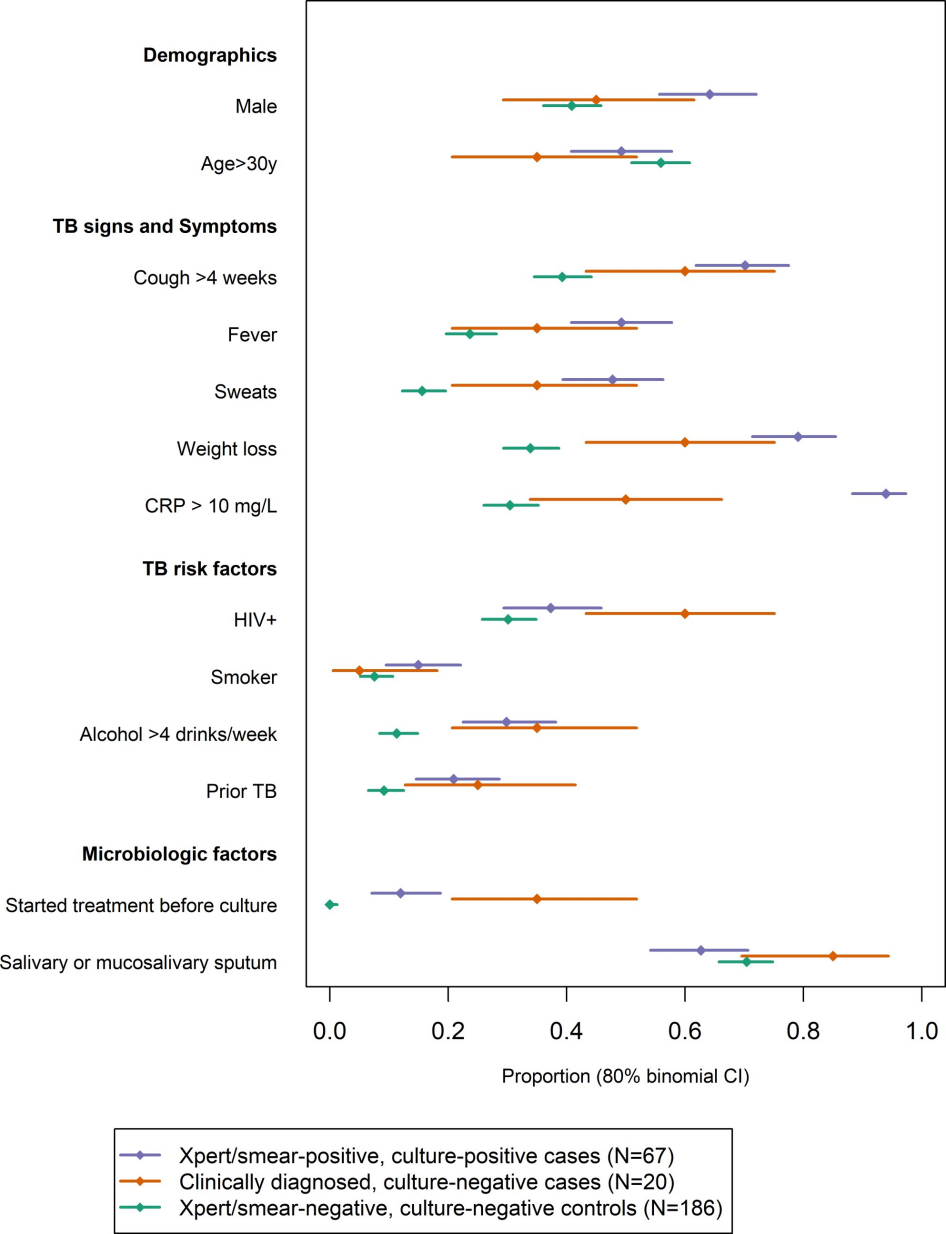
Key characteristics of patients with culture-concordant and culture-discordant clinical TB diagnoses. Error bars represent 80% binomial confidence intervals. Patients with negative evaluations (controls) but subsequent positive cultures are excluded, as are individuals with no culture result and the matched controls of cases with no culture result. Numerical values are also listed in S6 Table.

Fig 3 describes the 20 patients who were clinically diagnosed with TB but had negative sputum cultures, comparing them to patients with positive Xpert/smear and positive culture who were treated, and to control patients with negative Xpert/smear and negative culture who were not treated. Clinical diagnoses in culture-negative patients tended to be made in patients with TB risk factors (particularly HIV [RR = 1.6, 95% CI 1.0–2.6, compared to patients with positive Xpert/smear and confirmatory culture], although 40% were in HIV-negative individuals). Clinically-diagnosed, culture-negative patients also had fewer symptoms and lower CRP levels (including 8 [40%] with CRP < 2.5 mg/L which was extremely rare among culture-positive cases; S1 Fig).

Characteristics of patients with discrepant Xpert and culture results are shown in S2 Fig. All (6, 100%) patients with negative Xpert and positive culture were HIV-infected. Among those with positive Xpert and negative culture (9 patients, of whom 1 was empirically treated before their Xpert result became available), 100% had “low”" or “very low” semiquantitative TB detection by Xpert; 2 (22%) reported previous TB treatment.

Finally, empirically treated, culture-negative cases were more likely than other treated patients to have started treatment before they provided sputum to the research study for culture (RR = 2.4, 95% CI 1.1–5.2), but this treatment before culture was similarly common among culture-positive empirically treated patients (7 out of 20, 35%) and culture-negative empirically treated patients (2 out of 5, 40%) and may reflect the perceived urgency of treatment for those empirically treated.

## Discussion

The increasing worldwide use of Xpert and the ongoing transition to Xpert Ultra present the potential to reduce reliance on clinical diagnosis and to reduce empiric treatment for patients with negative bacteriologic results. The appropriate clinical response to the availability of on-site molecular TB testing depends on the performance of Xpert in a given patient population and clinicians’ ability to identify the remaining patients with TB who are missed by Xpert. In our evaluation of 300 presumptive pulmonary TB patients in an outpatient setting of high TB and HIV burden, we observed a high sensitivity and specificity for Xpert relative to sputum culture, such that very few (6/73, 8%) culture-confirmed TB cases were missed by Xpert. Clinical diagnosis and empiric treatment after negative Xpert identified two additional culture-confirmed TB cases, but at the expense of prescribing treatment to 18 patients with negative cultures for TB.

These results show that Xpert can perform as a useful diagnostic test in routine clinical care in a high-burden, low-resource setting, even before transition to the Ultra cartridge. Xpert provided bacteriologic diagnosis of over 90% of patients presenting with sputum-culture-positive TB, including over 80% of those with HIV coinfection. Ultra is expected to increase this sensitivity further, particularly for patients with HIV, although it may also reduce somewhat the high specificity that we observed [12]. In advocating for molecular TB diagnosis, however, it should be noted that practical obstacles limit full reliance on Xpert technology despite the low skill level required [13]. During our study, maintenance issues with auxiliary GeneXpert machine components (including the backup power supply and the results printer) forced health facilities to send specimens to external labs and caused several-day delays in Xpert results. Although nearly all patients had Xpert results within one week of presentation, these delays probably increased clinicians’ propensity to elect same-day empiric treatment for high-risk patients.

With Xpert performing accurately in practice and becoming increasingly available at the point of care in high-burden settings such as Uganda, it may be appropriate to reevaluate empiric treatment practices. Country-wide, Uganda’s non-bacteriologic TB diagnoses remained stable between 30 and 35% from 2013 to 2017 despite expanding Xpert use [14], consistent with the 26% of TB diagnoses that were made clinically in our study. Indeed, unless there has been an increase in patients with early, Xpert- and culture-negative patients seeking care, Xpert would be expected to increase bacteriologic diagnosis and thus reduce empiric treatment, even with no change in clinicians’ empiric treatment threshold. In contrast, the yield of clinical diagnoses has diminished: the probability that a clinically diagnosed, Xpert-negative patient had culture-positive TB was only 10% in our study, a considerable reduction compared to the 35% sputum culture positivity rate among empirically-treated Ugandan patients in the pre-Xpert era [1]. As the sensitivity of available diagnostic tests increases, false negative results become less likely, and it is reasonable to require stronger evidence before making a clinical diagnosis in the context of a negative test result. Our results suggest that clinicians may not have yet recalibrated their thresholds for empiric treatment to the Xpert context. Particularly as health systems transition further to Ultra’s higher sensitivity, clinicians may benefit from guidance on how to adapt their diagnostic intuition accordingly.

It is admittedly challenging to define how much empiric overtreatment is too much, and at what point the risks and costs of possible overtreatment outweigh the benefits of empiric treatment for a few patients. The 10% culture-positive rate that we observed among clinically-diagnosis patients is still four-fold higher than the probability of selecting a culture-positive individual by chance from our eligible Xpert-negative care-seeking study population (estimated at 2.5% based on a register review that suggested 6.0 eligible controls per enrolled case). Clinicians, even knowing that TB is unlikely in a given patient, may hesitate to defer treatment in patients with risk factors for high mortality or poor follow-up, or when they lack a treatable alternative diagnosis. It is also true that sputum TB diagnostics including culture are imperfect, that some of the patients with negative sputum cultures in our study may have had culture-negative TB disease that benefitted from empiric treatment, and that the judgment of experienced clinicians is a valuable tool for recognizing high-risk, sputum negative TB presentations. Notably, however, we found that most clinically-diagnosed, culture-negative patients were less sick than the typical TB case based on symptoms and inflammatory markers, suggesting treatment could have been deferred in these patients (e.g., for additional diagnostic data) with little expected harm. Jumping quickly to empiric TB treatment risks missing important non-TB diagnoses. In addition, in advanced HIV where clinicians may feel most compelled to treat empirically, emerging evidence shows a limited role for empiric TB treatment in reducing mortality [15].

Our study has some important limitations. Due to limited detail of clinical record-keeping and limited accessibility of patient records beyond the on-site diagnostic and treatment registers, we were unable to fully characterize the reasoning behind empiric treatment decisions. More extensive collection and expert review of data about empirically-treated, culture-negative patients–including the clinical evidence behind their diagnoses, the extent of their response to TB treatment, and the existence of alternative diagnoses–could help to elucidate the probability that such patients had true, culture-negative TB disease. In addition, although we have used sputum culture as a gold standard for diagnosis of pulmonary TB, we recognize that culture is imperfectly sensitive. Potential reasons for falsely negative sputum cultures include extra-pulmonary TB (less likely given predominately pulmonary symptoms among our culture-negative, clinically-diagnosed study participants), errors of laboratory processing, or antimicrobial treatment prior to culture. In our study, nearly one third of clinically diagnosed, culture-negative patients initiated treatment before sputum could be collected for culture, although most received no more than 3 days, which would be expected to reduce sputum CFU by less than one log [16] and to result in culture conversion at only the extreme of pauci-bacillary disease. A third limitation is that we did not collect data on the harms of missed diagnoses or of unnecessary treatments, and we are unable to quantitatively weight the risks and benefits of false-positive versus false-negative diagnoses.

In summary, judged against sputum culture, we observed high sensitivity and specificity of Xpert in routine outpatient care in urban Uganda, confirming its reliability as the primary test for TB diagnostic evaluation in similar settings of high TB and HIV prevalence. Empiric treatment remained common, at levels potentially comparable to the pre-Xpert era. Clinical diagnosis allowed a small number of additional culture-positive cases to be treated, but it had poor specificity in identifying such patients. As clinics increasingly use Xpert for TB diagnosis and transition to the Ultra cartridge, clinicians may need to adjust their threshold for empiric treatment, by continuing to treat Xpert-negative patients whose clinical presentations are most suggestive of TB, but by also more strongly pursuing possible alternative diagnoses in most Xpert-negative patients.

## Supporting information

S1 Fig **CRP measurements of clinically-diagnosed, culture-negative patients (shown in both panels, pink), compared to smear/Xpert-positive culture-positive cases (left panel only, green) and to smear/Xpert-negative culture-negative cases (right panel only, blue).** Lower and upper bounds of the assay used are 2.5 and 300 mg/L.
(TIF)

S2 Fig Comparison of patients with culture-concordant and culture-discordant Xpert results. (TIF)

S1 Table Distribution and agreement of liquid and solid culture results. (DOCX)

S2 Table Sensitivity and specificity of Xpert G4 and clinical judgment, relative to sputum culture (N = 239 patients tested using Xpert G4 cartridge). (DOCX)

S3 Table Sensitivity and specificity of Xpert Ultra and clinical judgment, relative to sputum culture (N = 41 patients tested using Xpert Ultra). (DOCX)

S4 Table Sensitivity and specificity of Xpert and clinical judgment, in sensitivity analysis that assumes all patients with missing or contaminated sputum cultures had a culture result consistent with their Xpert result. (DOCX)

S5 Table Sensitivity and specificity of Xpert and clinical judgment, in sensitivity analysis that assumes all patients with missing or contaminated sputum cultures had a culture result consistent with their clinicians’ treatment decisions. (DOCX)

S6 Table Key characteristics of patients with culture-concordant and culture-discordant clinical TB diagnoses (as shown graphically in Fig 3). (DOCX)
