# Supplementary material for: Empiric treatment of pulmonary TB in the Xpert era: Correspondence of sputum culture, Xpert MTB/RIF, and clinical diagnoses
Source: PLoS One. 2019 Jul 24;14(7):e0220251. doi: 10.1371/journal.pone.0220251 (PMC6655770; doi:10.1371/journal.pone.0220251)
Supplement: S1 Table — (DOCX) [file pone.0220251.s003.docx]

**S1 Table**

|  |  | Solid culture result | | | | |
| --- | --- | --- | --- | --- | --- | --- |
|  |  | Contaminated | Mycobacterial Growth | No Growth | Pending | Unable to produce sputum |
| Liquid culture result | Contaminated | 7 | 0 | 30 | 2 | 0 |
|  | MTBc Negative | 0 | 1 | 0 | 0 | 0 |
|  | MTBc Positive | 0 | 69 | 5 | 0 | 0 |
|  | No Growth | 3 | 0 | 176 | 6 | 0 |
|  | Pending | 0 | 0 | 0 | 1 | 0 |
|  | Unable to produce sputum | 0 | 0 | 0 | 0 | 1 |
