# Supplementary material for: Empiric treatment of pulmonary TB in the Xpert era: Correspondence of sputum culture, Xpert MTB/RIF, and clinical diagnoses
Source: PLoS One. 2019 Jul 24;14(7):e0220251. doi: 10.1371/journal.pone.0220251 (PMC6655770; doi:10.1371/journal.pone.0220251)
Supplement: S2 Table — (DOCX) [file pone.0220251.s004.docx]

## **S2 Table**

|  | Sensitivity | | | Specificity | | |
| --- | --- | --- | --- | --- | --- | --- |
|  | **N** | **Estimate** | **95% CI** | **N** | **Estimate** | **95% CI** |
| Xpert G4, All patients | 61/64 | 95% | 87-99% | 167/175 | 95% | 91-98% |
| Xpert G4, HIV+ | 22/25 | 88% | 69-97% | 53/55 | 96% | 87-100% |
| Xpert G4,  Previously treated | 13/13 | 100% | 75-100% | 16/18 | 89% | 65-99% |
| Xpert G4 + clinical diagnosis, All patients | 63/64 | 98% | 92-100% | 150/175 | 86% | 80-91% |
| Xpert G4 + clinical diagnosis, HIV+ | 24/25 | 96% | 80-100% | 42/55 | 76% | 63-87% |
| Xpert G4 + clinical diagnosis, Previously treated | 13/13 | 100% | 75-100% | 13/18 | 72% | 47-90% |
