# Supplementary material for: Empiric treatment of pulmonary TB in the Xpert era: Correspondence of sputum culture, Xpert MTB/RIF, and clinical diagnoses
Source: PLoS One. 2019 Jul 24;14(7):e0220251. doi: 10.1371/journal.pone.0220251 (PMC6655770; doi:10.1371/journal.pone.0220251)
Supplement: S3 Table — (DOCX) [file pone.0220251.s005.docx]

## **S3 Table**

|  | Sensitivity | | | Specificity | | |
| --- | --- | --- | --- | --- | --- | --- |
|  | N | Estimate | 95% CI | N | Estimate | 95% CI |
| Xpert Ultra, All patients | 6/9 | 67% | 30-93% | 31/32 | 97% | 84-100% |
| Xpert Ultra, HIV+ | 3/6 | 50% | 12-88% | 13/14 | 93% | 66-100% |
| Xpert Ultra, Previously treated | 1/1 | 100% | 3-100% | 3/4 | 75% | 19-99% |
| Xpert Ultra + clinical diagnosis, All patients | 6/9 | 67% | 30-93% | 30/32 | 94% | 79-99% |
| Xpert Ultra + clinical diagnosis, HIV+ | 3/6 | 50% | 12-88% | 12/14 | 86% | 57-98% |
| Xpert Ultra + clinical diagnosis, Previously treated | 1/1 | 100% | 3-100% | 3/4 | 75% | 19-99% |
