# Supplementary material for: Empiric treatment of pulmonary TB in the Xpert era: Correspondence of sputum culture, Xpert MTB/RIF, and clinical diagnoses
Source: PLoS One. 2019 Jul 24;14(7):e0220251. doi: 10.1371/journal.pone.0220251 (PMC6655770; doi:10.1371/journal.pone.0220251)
Supplement: S4 Table — (DOCX) [file pone.0220251.s006.docx]

## **S4 Table**

|  | Sensitivity | | | Specificity | | |
| --- | --- | --- | --- | --- | --- | --- |
|  | **N** | **Estimate** | **95% CI** | **N** | **Estimate** | **95% CI** |
| Xpert, All patients | 68/74 | 92% | 83-97% | 209/218 | 96% | 92-98% |
| Xpert, HIV+ | 25/31 | 81% | 63-93% | 73/76 | 96% | 89-99% |
| Xpert  Previously treated | 14/14 | 100% | 77-100% | 23/26 | 88% | 70-98% |
| Xpert + clinical diagnosis, All patients | 70/74 | 95% | 87-99% | 190/218 | 87% | 82-91% |
| Xpert + clinical diagnosis, HIV+ | 27/31 | 87% | 70-96% | 60/76 | 79% | 68-87% |
| Xpert + clinical diagnosis, Previously treated | 14/14 | 100% | 77-100% | 19/26 | 73% | 52-88% |
