# Supplementary material for: Empiric treatment of pulmonary TB in the Xpert era: Correspondence of sputum culture, Xpert MTB/RIF, and clinical diagnoses
Source: PLoS One. 2019 Jul 24;14(7):e0220251. doi: 10.1371/journal.pone.0220251 (PMC6655770; doi:10.1371/journal.pone.0220251)
Supplement: S5 Table — (DOCX) [file pone.0220251.s007.docx]

## **S5 Table**

|  | Sensitivity | | | Specificity | | |
| --- | --- | --- | --- | --- | --- | --- |
|  | **N** | **Estimate** | **95% CI** | **N** | **Estimate** | **95% CI** |
| Xpert, All patients | 68/75 | 91% | 82-96% | 208/217 | 96% | 92-98% |
| Xpert, HIV+ | 25/32 | 78% | 60-91% | 72/75 | 96% | 89-99% |
| Xpert,  Previously treated | 14/15 | 93% | 68-100% | 22/25 | 88% | 69-97% |
| Xpert + clinical diagnosis, All patients | 71/75 | 95% | 87-99% | 190/217 | 88% | 82-92% |
| Xpert + clinical diagnosis, HIV+ | 28/32 | 88% | 71-96% | 60/75 | 80% | 69-88% |
| Xpert + clinical diagnosis, Previously treated | 15/15 | 100% | 78-100% | 19/25 | 76% | 55-91% |
