# Supplementary material for: Empiric treatment of pulmonary TB in the Xpert era: Correspondence of sputum culture, Xpert MTB/RIF, and clinical diagnoses
Source: PLoS One. 2019 Jul 24;14(7):e0220251. doi: 10.1371/journal.pone.0220251 (PMC6655770; doi:10.1371/journal.pone.0220251)
Supplement: S6 Table — (DOCX) [file pone.0220251.s008.docx]

**S6 Table**

|  | Xpert/smear-positive, culture-positive cases (N=67) | Clinically diagnosed, culture-negative cases (N=20) | Xpert/smear-negative, culture-negative controls (N=177) |
| --- | --- | --- | --- |
| Salivary or mucosalivary sputum | 63% (54-71%) | 85% (70-94%) | 70% (66-75%) |
| Started treatment before culture | 12% (7-19%) | 35% (21-52%) | 0% (0-1%) |
| Prior TB | 21% (15-29%) | 25% (13-41%) | 9% (7-12%) |
| Alcohol >4 drinks/week | 30% (22-38%) | 35% (21-52%) | 11% (8-15%) |
| Smoker | 15% (9-22%) | 5% (1-18%) | 8% (5-11%) |
| HIV+ | 37% (29-46%) | 60% (43-75%) | 30% (26-35%) |
| CRP > 10 mg/L | 94% (88-97%) | 50% (34-66%) | 30% (26-35%) |
| Weight loss | 79% (71-85%) | 60% (43-75%) | 34% (29-39%) |
| Sweats | 48% (39-56%) | 35% (21-52%) | 16% (12-20%) |
| Fever | 49% (41-58%) | 35% (21-52%) | 24% (20-28%) |
| Cough >4 weeks | 70% (62-78%) | 60% (43-75%) | 39% (34-44%) |
| Age>30y | 49% (41-58%) | 35% (21-52%) | 56% (51-61%) |
| Male | 64% (56-72%) | 45% (29-62%) | 41% (36-46%) |
